# Whose side are you on? Complexities arising from the non-combatant status of military medical personnel

**DOI:** 10.1007/s40592-022-00168-2

**Published:** 2023-01-11

**Authors:** Michael C. Reade

**Affiliations:** Medical School, University of Queensland, Brisbane, QLD Australia

**Keywords:** Conflict of interest, Ethics, Human rights, Military health, Military medicine

## Abstract

Since the mid-1800s, clergy, doctors, other clinicians, and military personnel who specifically facilitate their work have been designated “non-combatants”, protected from being targeted in return for providing care on the basis of clinical need alone. While permitted to use weapons to protect themselves and their patients, they may not attempt to gain military advantage over an adversary. The rationale for these regulations is based on sound arguments aimed both at reducing human suffering, but also the ultimate advantage of the nation-state fielding non-combatant staff. However, this is sometimes not immediately apparent to combatant colleagues. Clinicians in the armed force are also military officers, owing a “dual loyalty” that can create conflict if their non-combatant status is not well understood. Historical examples of doctors breaching their responsibilities include prioritisation of combat capability over the rights of individual soldiers (as occurred when scarce medical resources were allocated to soldiers more likely to return to battle in preference to those most likely to die without them), use of physicians to facilitate prisoner interrogation, medical research or treatment to enhance physical performance at the expense of health, application of Medical Rules of Eligibility according to factors other than clinical need, provision of treatment contingent upon support for military objectives, and use of medical knowledge to enhance weapons. However, not being a combatant party to a conflict does not imply that the non-combatant clinician cannot act in the national interest. Indeed, by adhering to the same universal ethics as their civilian colleagues, military clinicians provide optimal care to their own troops, facilitate freedom of action in host nations, and build positive international relationships during the conflict and in the post-conflict state.

## Introduction

The Australian Defence Force (ADF), in common with the armed forces of the other 195 nations that have ratified the Geneva Conventions, recognises its medical staff as “non-combatants” accorded specific rights and responsibilities. Medical officers (doctors) share this designation with religious personnel and with others whose primary role is to care for the sick and wounded. This includes not only other clinicians, such as nurses and allied health practitioners, but also non-clinical staff in support roles such as health planners, biomedical technicians and drivers of ambulances. Non-combatant status is designated by one of the distinctive emblems defined in Article 38 of the First Geneva Convention (ICRC 1949a): the Red Cross, Red Crescent or Red Crystal, although not displaying one of the Distinctive Emblems does not deprive a person, building or vehicle of protected status. The Red Cross was derived from the reversed colours of the Swiss flag, reflecting the national origin of the Convention. It was not intended to convey any Christian significance. (Wilkins and Dieppe 2017) Nor is it intended to imply a healthcare function: only popular culture has misappropriated the Red Cross to signify first aid or medicine, (Slim 1989) and it is worth noting that in order to avoid this confusion the official emblems of civilian ambulance services specifically should not incorporate a Red Cross, unless also claiming non-combatant status in a conflict zone.

The four Geneva Conventions and their three Additional Protocols state that non-combatants must not participate directly in hostile action. (Wilkins and Dieppe 2017) Their sole function within the Area of Operations is to provide (or facilitate) medical care to the sick and wounded, and they must do this only on the basis of clinical need rather than the affiliation of the patient. They must not be hindered in this task by any party to the conflict, and they cannot become Prisoners of War. They are permitted to carry weapons but may only use these to protect themselves or their patients, not to gain military advantage over an adversary. To attempt to do so while claiming the protection afforded a non-combatant would be a breach of the Laws of Armed Conflict (a “war crime”), as specified in Article 37 of Additional Protocol 1 (ICRC 1977a). This Article prohibits killing, injuring or capturing an adversary by resort to “perfidy”, defined as “inviting the confidence of an adversary to lead him to believe that he is entitled to, or is obliged to accord, protection under the rules of international law applicable in armed conflict, with intent to betray that confidence”, such as would occur by “the feigning of protected status by the use of signs, emblems or uniforms”.

While the rationale for the criteria listed above are based on sound arguments aimed both at reducing overall human suffering in times of conflict and also at ultimate advantage to the nation-state fielding the non-combatant staff, practical application of these precepts is sometimes problematic. In particular, the advantage to one’s own side that is gained from impartial provision of medical treatment is not always immediately apparent to combatant colleagues. The discussion presented in this paper takes the practical viewpoint of a military medical officer presented with situations in which there is an apparent conflict between duty to their professional ethics as a doctor, and their duty as a military officer. Choosing the perspective of a medical officer over that of other non-combatants is largely arbitrary; the principles discussed apply equally to priests, nurses, and others, but as most of the published historical examples have focussed on doctors, for simplicity that is the most straightforward approach. Through these examples, the complexity of what has been termed “dual loyalty” will be illustrated. Such examples are not confined to the treatment of enemy combatants. There can be conflict between the welfare of individual patients of one’s own side and the goals of the organisation. Furthermore, interpretation of prohibition against “hostile action” is sometimes not straightforward. However, in each of these cases, application of the over-riding principles of non-combatant status, articulated in more detail in following sections, will be seen to provide sufficient guidance. Implementation of these principles requires permeation of sound ethical principles throughout a military organisation, and in particular requires senior military medical leadership with sufficient experience and influence to ensure practising clinicians are adequately supported. Examples of when this has, and has not, occurred illustrate the need for constant vigilance.

## Historical context

The noble goals of the Geneva Conventions as they relate to non-combatants are a relatively modern construct. Only in 1864 did the International Committee of the Red Cross gain Swiss Government support for a conference of twelve European countries that ultimately drafted the first ten Articles of what were to become the Geneva Conventions. (Wilkins and Dieppe 2017) The first two of these Articles recognised ambulances, hospitals and their staff as neutral in conflict. Most published accounts of military medical practice before this time focus on technical clinical details rather than questions of ethics, and in particular make scant mention of how enemy combatants were treated. However, there are suggestions from the actions of several prominent doctors that they did not consider themselves in the modern non-combatant construct.

In the 5th century BC, Hippocrates required that physicians “apply … measures for the benefit of the sick according to … ability and judgement; (keeping) them from harm and injustice” (Hajar 2017). To imagine that this requirement extended to all people – even enemy soldiers - is sometimes claimed, (Benton and Atshan 2016) but is questioned by the reply Hippocrates is said to have given to Artaxerxes, King of Persia, when requested to treat Persian soldiers suffering from plague: “Tell your master I am rich enough; honor will not permit me to succor the enemies of Greece”. (Sidel and Levy 2003) In the Christian tradition, too, the Knights Hospitallers of St John of Jerusalem began as a religious order in the 11th century, in part to care for sick and poor Christian pilgrims to the Holy Land. However, in practice they became a formidable military presence of “warring physicians”, defending Christian territory against Muslim invasion. (Sidel and Levy 2003).

Even in modern history, prominent doctors have found no inconsistency between their roles as healers and as combatants. Dr John Crimmin was a Surgeon in the Bombay Medical Service. During the Burma campaign in 1889, near Lwekaw in eastern Karenni, while attached to the 27th Bombay Infantry, Dr Crimmin fought off enemy soldiers whilst attending wounded men, but also “joined the fighting line … very shortly afterwards they were engaged in driving the enemy from small clumps of trees and bamboo, in which the Karens took shelter”. (Starling 2009) While defending himself and his patients was entirely consistent with the modern concept of a non-combatant, engaging in the subsequent battle arguably was not. Dr Crimmin was awarded the Victoria Cross for these actions, subsequently served on the North West Frontier during the First World War, and was appointed Honorary Physician to the King in 1916. Citations for the Victoria Cross awarded to other British doctors show similar blurring of the distinction between defensive and combatant action. (‘The London Gazette’ 1861) Even clearer examples occur in US military history. Dr Leonard Wood (1860–1927) earned the Medal of Honor for actions during the 1886 Indian Wars. While serving as an Assistant Surgeon to the US Army in Arizona, he volunteered to carry dispatches 100 miles through enemy territory, and later commanded soldiers of the 8th Infantry Regiment after all their officers had been killed in the pursuit of the Apache leader Geronimo. While he went on to be personal physician to two US presidents, his later career was spent primarily in administrative, political and military appointments, including as the US Army Chief of Staff. Similarly, in 1861 Dr Bernard Irwin, also an Assistant Surgeon, took command of US Army soldiers in Arizona, also earning the Medal of Honor. (Sidel and Levy 2003) During the US Civil War, Jacob Raud, an Assistant Surgeon with the 210th Pennsylvania Infantry, in 1865 “discovering a flank movement by the enemy at Hatcher’s Run, Virginia, appraised the commanding general at great peril, and though a noncombatant voluntarily participated with the troops in repelling this attack.”, for which he, too, was awarded the Medal of Honor. (Sidel and Levy 2003) Clearly the notion that medical personnel must not participate in combat but rather perform clinical work without favour for the benefit of patients on all sides of a conflict is relatively modern concept. Why might this notion have evolved, and does it ultimately offer more benefit to a country than would be true of clinicians freed from civilian medical ethics to adopt a partisan affiliation?

## Origins of the non-combatant clinician

The conceptual origin of the non-combatant clinician appears coincident with the foundation of the International Committee of the Red Cross in 1863. The precipitant to this event was the observation by businessman Henry Dunant (1828–1910) of the aftermath of the battle of Solferino in 1859, which he documented in an 1862 book (*“Un souvenir de Solférino” / “A Memory of Solferino” (*Dunant 1959*)*) that he subsequently promoted with considerable success, leading to sufficient international government support to facilitate the agreement on the first Geneva Conventions. Dunant himself then essentially retired from public life, declaring bankrupt and living in poverty on the generosity of friends and relatives until his death. His ideas, however, had sufficient strength to live on without his personal support; in 1901 he was recognised with the first Nobel Peace Prize.

Dunant’s own personal actions in Solferino were to establish the tradition of the impartial non-combatant medical attendant. He recruited a team of assistants of every nationality present – French, Austrian, Italian, German, English, Canadian and others - to care for the 30,000 wounded French and Austrian soldiers. He particularly highlighted the efforts of French military surgeons in treating enemy Austrian patients, and in the value of captured Austrian doctors who cared for French casualties. (Dunant 1959) At the conclusion of his account, he asks “why have I told of these scenes of pain and distress, and perhaps aroused painful emotions? …. Would it not be possible, in time of peace, to form relief societies for the purpose of having care given to the wounded in wartime by zealous, devoted and thoroughly qualified volunteers”. Dunant recognised that for such “societies” to be effective their work would have to be facilitated by the military forces of each combatant nation, but asked “is there a military commissary … who would not be grateful?”.

While the British Army was accompanied by Regimental Surgeons as early as 1660, it was not until the formation of the Medical Staff Corps in 1855 that any systematic large-scale attempt was made to provide care for sick and wounded soldiers. However, even after the formation of the Royal Army Medical Corps in 1898, the military organisation of healthcare remained so poor that even throughout the First World War (‘History of the Royal Army Medical Corps’ 2022), civilian organisations (along the lines envisaged by Dunant) were relied upon. (‘History of the Royal Army Medical Corps’ 2022) Prominent examples included Florence Nightingale’s volunteer nurses working at the British military hospital at Scutari (1854–1856) during the Crimean War, the principles of which were unchanged in the work of the Red Cross Voluntary Aid Detachments, and the “Volunteer Hospitals”, during the First World War. (Martin 2002).

The advent of manoeuvre warfare in the Second World War meant that reliance on volunteer organisations was no longer acceptable in the deployed environment. Armed forces required medical support that could be integrated with a rapidly moving battle, and which could be relied upon as a direct command element. The role of the International Committee of the Red Cross during that conflict was primarily to advocate for acceptable treatment for Prisoners of War, displaced persons and refugees. (Dunant 1959) However, the principles of impartial non-combatant military healthcare established during its period of co-existence with the voluntary aid organisations persisted. In part, this may have been continuation of a tradition without explicit consideration. What consideration might have occurred has not survived to readily accessible academic literature. However, as will be argued below, there are compelling reasons to preserve this non-combatant tradition. Before exploring these in detail, a more comprehensive presentation of the legal framework currently governing this topic is warranted.

## Elements of the Geneva conventions and their protocols specifying the nature of non-combatant status of military medical personnel

The first three Geneva Conventions of 1949 (ICRC 1949a, 1949b, 1949c) and their three Additional Protocols adopted in 1977 (ICRC 1977a, 1977b) and 2005 (ICRC 2005) contain Articles pertaining to the non-combatant status of military medical personnel. The fourth Geneva Convention (ICRC 1949d) relates to the protection of civilians in wartime and so is not relevant to this discussion. Table 1 summarises the relevant Articles and is presented to provide a comprehensive overview. An interesting and often overlooked point is contained in Article 22 of Convention 1, (1949) which lists amongst conditions that do not deprive a medical establishment of its non-combatant status “That personnel and material of the veterinary service are found in the unit or establishment, without forming an integral part thereof”. The clear implication is that veterinarians must not be an integral part of the protected establishment. The treatment of animals (such as horses and military working dogs) was presumably characterised by those who framed the Article as being of assistance to the war effort, in the same manner as the repair of other military equipment, rather than as treatment of living entities to be accorded protection in a manner equivalent to wounded or sick people.

**Table 1:** Summary of the 1949 Geneva Conventions and their 1977 and 2005 Additional Protocols relevant to the non-combatant status of military medical personnel (from (Wilkins and Dieppe 2017)) ***The numbers in the “Article” column of this table need to align with the relevant “title” and “requirement” in the next two columns, as they did in the submitted manuscript. As presented in this table and in the pdf, the table in this format makes no sense I have tried to fix this myself but the online system does not allow the table to be edited***

Convention or Protocol	Article	Title	Requirement
Geneva Convention (I) 1949 For the Amelioration of the Condition of the Wounded and Sick in Armed Forces in the Field (1949a)	19	Protection of medical units and establishments	Medical establishments shall be protected. They must not be situated valid near military targets.
	20	Protection of hospital ships	Hospital ships shall not be attacked from land
	21	Discontinuance of protection of medical establishments and units	Protection will cease if used for acts harmful to the enemy, after a warning is given
	22	Conditions not depriving medical units of protected status	Medical personnel may use arms to defend themselves and their patients
	24	Protection of permanent Personnel	Medical & related staff shall be protected in all circumstances
	25	Protection of Auxiliary Personnel	Personnel carrying out medical & related duties shall be protected while doing so.
	28	Retained Personnel	Medical & related staff in the hands of the enemy are not prisoners of war. They can continue to work, and must only be retained if required to treat prisoners of war from their own side of the conflict.
	30	Return of Medical & related personnel	Medical & related staff in the hands of the enemy not required to perform tasks under Article 28 must be returned to their own side.
	40	Identification	Medical personnel will carry an identity card and wear a distinctive armlet on the left arm
Geneva Convention (II) 1949 For the Amelioration of the Condition of the Wounded, Sick and Shipwrecked Members of the Armed Forces at sea (ICRC 1949b)	22	Hospital ships	Hospital ships shall be notified to all combatants and protected
	23	Protection of medical establishments ashore	Shore medical establishments shall not be attacked from the sea
	28	Protection of sick bays	Sick bays must be protected so long as they are required for the care of the sick
	30	Employment of hospital ships	Hospital ships shall assist wounded, sick and shipwrecked without distinction of nationality
	34	Discontinuance of protection of hospital ships	Protection will cease if used for acts harmful to the enemy, after a warning is given. Hospital ships may not possess any secret wireless (or similar) code.
	36	Protection of personnel of hospital ships	Medical & related staff, and hospital ship crews, shall be protected, whether or not there are patients on board
	37	Medical and religious personnel of other ships	The medical & related staff shall, if they fall into enemy hands, be protected.
	42	Identification	Medical personnel will carry an identity card and wear a distinctive armlet on the left arm
Geneva Convention (III) 1949 Relative to the Treatment of Prisoners of War (ICRC 1949c)	33	Rights and privileges of retained personnel	Medical & related staff are not considered prisoners of war when captured by the enemy.
Additional Protocol (I) 1977 Relating to the Protection of Victims of International Armed Conflicts (ICRC 1977a)	10	Protection and care	All wounded and sick, to whichever Party they belong, shall receive, to the fullest extent practicable, the medical care and attention required by their condition
	16	General protection of medical duties	“Under no circumstances shall any person be punished for carrying out medical activities compatible with medical ethics, regardless of the person benefiting therefrom”
Additional Protocol (II) 1977 Relating to the Protection of Victims of Non-International Armed Conflicts (ICRC 1977b)	7	Protection and care	All wounded & sick, whether or not they have taken part in the armed conflict, shall receive, to the fullest extent practicable and with the least possible delay, the medical care and attention required by their condition
	9	Protection of medical and religious personnel	Medical and religious personnel shall be protected and helped in their duties. They shall not be compelled to carry out tasks which are not compatible with their humanitarian mission. Medical personnel may not be required to give priority to any person except on medical grounds.
	10	General protection of medical duties	“Under no circumstances shall any person be punished for having carried out medical activities compatible with medical ethics, regardless of the person benefiting therefrom”
	11	Protection of medical units and transports	Medical units shall not be the object of attack
	12	The distinctive emblem	The red cross, red crescent or red lion shall be displayed by medical and religious personnel and medical units
Additional Protocol (III) 2005 Relating to the Adoption of an Additional Distinctive Emblem (ICRC 2005)	2	Distinctive emblems	Introduces the Red Crystal as the third Distinctive Emblem, in addition to the Red Cross and the Red Crescent

Several points are worthy of particular note in the context of this discussion. Both Additional Protocol I and II (ICRC 1977a, 1977b) require that “Under no circumstances shall any person be punished for carrying out medical activities compatible with medical ethics, regardless of the person benefiting therefrom”. Additional Protocol I further elaborates: “Persons engaged in medical activities shall not be compelled to perform acts or to carry out work contrary to the rules of medical ethics or to other medical rules designed for the benefit of the wounded and sick or to the provisions of the Conventions or of this Protocol, or to refrain from performing acts or from carrying out work required by those rules and provisions”. In essence, these Additional Protocols dictate that professional medical ethics must take priority over any other legal or ethical requirement that might be assigned to the clinician, including any requirement that might arise from their status as a military officer. Both Additional Protocols also regulate information received in the context of clinical care. In Additional Protocol I: “No person engaged in medical activities shall be compelled to give to anyone belonging either to an adverse Party, or to his own Party except as required by the law of the latter Party, any information concerning the wounded and sick who are, or who have been, under his care, if such information would, in his opinion, prove harmful to the patients concerned or to their families”. Additional Protocol 2 contains a similar statement, accompanied by an equivalent legal caveat: “The professional obligations of persons engaged in medical activities regarding information which they may acquire concerning the wounded and sick under their care shall, subject to national law, be respected”. The provision that confidentiality of information is “subject to national law” contradicts, to a degree, the all-encompassing ethical prioritisation of the first statement.

While the 1949 Geneva Conventions have been ratified by 196 nations, the Additional Protocols have not. Only 168 States are party to Additional Protocol I and 164 States to Additional Protocol II. A notable exception is the United States, which has signed but not ratified these Protocols. (Blokina and Jurkowski 2019) The stated reasons for this were complex but were primarily related to concerns over what are considered to be legitimate military targets, and also the possible recognition of terrorist groups as equivalent to the armed forces of nation-states. (Reagan 1987) The provisions in Additional Protocols I and II requiring equal treatment of civilians, discussed in detail below, were not cited as reasons.

## The problem of “dual loyalty”

The 1977 Additional Protocols to the Geneva Conventions make it clear that a military doctor is expected to conform to the same ethical principles as every other doctor. Soldiers should surely be comforted that their care is not in the hands of a form of quasi-doctor or other clinician. However, doctors and other clinicians in most modern armed forces are also military officers, expected to display loyalty to their commanders, institution, and nation. Mostly, these two roles are not in conflict. Even if the role of the Royal Australian Army Medical Corps (and similar institutions worldwide) is “to contribute to the Army’s operational capability through the conservation of manpower by promoting health and well-being, through the prevention of disease and injury, and through the care, treatment and evacuation of sick and wounded”, (‘Royal Australian Army Medical Corps’ 2021) rather than to prioritise the welfare of individual soldiers, in practice in all but the most extreme circumstances the military also has the best interests of its members at heart. Further, while many academic discussions characterise military forces in negative terms in opposition to medicine acting for good, in ideal circumstances the military force to which a clinician belongs is working towards a just and ethically sound purpose. (Rochon 2015) However, history instructs that occasionally this is not the case. When these roles do come into conflict, as Eagan has noted, (Eagan 2019) there is not only a problem of divided loyalty, but a fundamental incompatibility in the cultures of medicine (in particular amongst the clinical professions) and that of the Profession of Arms.

Physicians see themselves as members of a profession that derives its identity and status from group adherence to ethical principles and standards of behaviour that have evolved over many centuries, and which transcend nationality, socioeconomic status and politics. Deviation from what is considered acceptable conduct is followed by swift expulsion from the professional group. Essential to the practice of medicine is personal responsibility for professional mastery, the primacy of individual patient interests (for example, the UK General Medical Council requires, “as a good doctor you will make the care of your patient your first concern” (GMC(UK) 2019)), and the requirement to exercise one’s own judgement and skill in professional practice rather to abrogate responsibility to a higher authority. For these reasons, patients are commonly encouraged to trust their doctor as their personal agent rather than as the representative of a healthcare system. The importance of the individualised doctor-patient relationship is respected even in nationalised healthcare systems that seek to standardise care to equalise patient outcomes and reduce costs.

The Profession of Arms has a similarly strong cultural understanding of what it means to be a military officer or Other Rank, but in some respects this culture fundamentally conflicts with that of a doctor. While military officers have a responsibility to think for themselves to discern what is in the national interest in order to carry out the responsibilities assigned by their commissions, in practice military culture values loyalty, obedience, camaraderie and team cohesion. Exercise of personal judgement, for example in routinely questioning the merits of instructions, is less valued than following orders or (at best) using initiative to meet a commander’s intent. As an illustrative example, the Australian Army’s “Contract with Australia” states in part: “I am committed to learning and working for the team; I believe in trust, loyalty and respect for my Country, my mates and the Army”. (Army 2021).

When circumstances do arise in which there is a practical conflict between a doctor’s obligations to a patient (or to professional ethical standards more broadly) and to the military, as will be seen, the cultural incompatibility identified here can magnify the problem to the point that resolution is impossible.

## Classification and examples of problems arising from “dual loyalty”

Table 2 classifies practical examples of conflict between a clinician’s ethical responsibilities to their profession, and those they owe to their employer. Several illustrative examples have reached prominence.

**Table 2:** Classification of conflicts between professional medical ethics and medical responsibilities (after (Rochon 2015) and other sources)

Classification of ethical conflict	Example
Inability to prioritise individual patient’s interest over institutional goals	• Inability to maintain patient confidentiality • Prioritisation of public health measures (e.g. use of vaccinations or nerve agent prophylaxis) over individual patient autonomy • Rationing of scarce medical resources according to military rather than clinical needs • Certification as “fit to fight” • Certification as “fit to interrogate” • Facilitation of interrogation • Failure to report mistreatment • Facilitation of force-feeding of prisoners • Medical research or treatment to enhance performance at the expense of health
Artificial constraints on the extent or type of treatment able to be provided	• Application of Medical Rules of Eligibility • Triage of patients of different nationalities within the Medical Rules of Eligibility
Obligation to support the war effort	• Making provision of medical care to a host nation contingent upon support • Participation in interrogation of prisoners • Medical research to enhance weapons

On 14 September 2003, British soldiers arrested Baha Mousa, a 26-year old father of two in Basra, Iraq. During his subsequent 36 h in custody in the care of the 1st Battalion, Queen’s Lancashire Regiment, he sustained 93 separate injuries that ultimately led to his death. (Gage 2011) Captain Derek Keilloh, the battalion’s Regimental Medical Officer, made an unsuccessful attempt at resuscitation after Mr Mousa had suffered a cardiac arrest. The subsequent inquiry found that Dr Keilloh’s attempted resuscitation was technically proficient. However, he was a junior medical officer who had received “no previous training or experience of dealing with prisoners of war or civilian detainees”, had been “ordered on three days’ notice to transfer to Iraq. He was given no time to acclimatise to conditions”, and “his two Senior Medical Officers rarely got in touch with him, which contributed to him having a strong feeling of isolation”. He was “not criticise(d) … for adopting the procedures which he inherited” in not routinely examining detainees. The enquiry recognised that he had been placed in a very difficult situation in an infantry unit with a strong organisational culture that would have been very difficult to disrupt, especially acting in isolation from any (medical) professional support. Nonetheless, the inquiry found “He ought to have instituted a system by which on entry to the (detention facility) all detainees were examined by himself or one of his senior medics”, and that subsequent to Mr Mousa’s death “it (was) very difficult to believe that Keilloh did not see signs of mistreatment on Baha Mousa’s body and that he had no recollection of any discussion about injuries seen by others”. (Gage 2011) On the basis of his failure to act in the best interests of detainees deemed to be his patients in the 36 h prior to Mr Mousa’s death, and more importantly due to the charge of subsequently providing dishonest evidence about the injuries he was believed to have observed on Mr Mousa (presumably in an attempt to protect those responsible), in 2012 the UK General Medical Council ruled Dr Keilloh unfit to practice medicine. (Cobain 2012) While no-one could argue that being complicit in torture was part of Captain Keilloh’s proper military duty, it can only be assumed that his deviation from professional medical ethics (and indeed actions specifically required by the 1975 Declaration of Tokyo (WMA 1975)) was motivated in part by the (perhaps impossible) situational conflict that arose when seemingly he, alone, was left responsible for advocating for a patient’s welfare in a system that had become morally corrupted.

The involvement of US military doctors in the interrogation of prisoners during the “War on Terror”, 2001-present, has been the subject of great controversy. (Annas 2008) US military physicians were reportedly ordered to force-feed hunger striking prisoners held in detention in Guantanamo Bay, with the rationale that even if hunger striking is a form of asymmetric warfare (and hence combating this would be advancing the war effort) and violates the requirement for informed consent, these prisoners lacked the capacity to refuse consent either due to physical incapacity or peer pressure. US military physicians were reportedly also required to certify prisoners as “fit for interrogation”, and a 2006 US Department of Defense instruction authorised physicians to certify prisoners as fit for “punishment” and even to administer the punishment if it was “in accordance with applicable law”. (Annas 2008) In response to American Medical Association objection to these policies, the US Department of Defense was noted to have provided advice that in essence required its physicians not follow nationally and internationally accepted medical ethics (including, for example, the explicit requirements of the 1975 Declaration of Tokyo related to the treatment of prisoners (WMA 1975)), even though its written guidance provided contradictory advice: all military physicians were to “regularly monitor their behavior and remain within professional ethical boundaries as established by their professional associations, by the licensing State, and by the military.” (Annas 2008).

The post-2001 deployments of military hospitals to Afghanistan and Iraq presented another problem to many military doctors – having to abide by, and in some cases interpret, Medical Rules of Eligibility. Article 10 of Additional Protocol I, and Article 7 of Additional Protocol II, state that medical care must be provided to all wounded and sick, “whether or not they have taken part in the armed conflict”, “to the fullest extent practicable” (Table 1). One US physician argued that, by not having ratified Additional Protocols I and II, US physicians were not obliged to provide the same standard of healthcare to host-nation civilians and combatants as they did to US service personnel. (Lounsbury 2004) Despite this, for any Iraqi admitted to his hospital, this physician reported that the same standard of emergency care was provided; only the post-acute care following discharge differed, as Iraqis were not eligible for evacuation to the United States. Although well-resourced to treat combat casualties, modern military hospitals deployed to Iraq, Afghanistan and elsewhere have been clearly inadequate to take over the role of the host nation medical system, and from a health development perspective it would have been highly inappropriate for them to have done so. During much of the conflict, it was common practice for civilians injured as a result of coalition military activity to be eligible for life-saving treatment but not complex rehabilitation, and civilians were usually not eligible for non-trauma related care. Applying these rules was usually not left to hospital clinicians. Rather, Medical Rules of Eligibility were applied prehospital by non-clinicians. Once a patient arrived in a coalition military hospital they were treated the same as any other patient. The theoretical problem of having to choose between a wounded coalition soldier and a host-nation civilian, although this reportedly did occur, (Howe 2015) fortunately only arose infrequently, in part (once again) due to thoughtful prehospital decisions that did not result in mixtures of casualties arriving simultaneously in the one facility. In this way, the military system protected its non-combatant clinicians from some of the ethical difficulties that Medical Rules of Eligibility might otherwise have presented.

The opposite problem – being compelled to provide care to a host nation population in circumstances that make this inappropriate – has also occurred. Inappropriate circumstances have ranged from establishing Medical Assistance Clinics providing limited primary care with little or no possibility of follow-up (clinically poor medicine, but not in violation of the Geneva Conventions), to attempting to make the provision of medical care contingent on the support of the host nation. Examples date from the Vietnam War (Wilensky 2004) to post-2001 Afghanistan. (Rochon 2015) One prominent example of an attempt to prevent this type of work was that of Dr Howard Levy, a dermatologist conscripted into the US Army during the Vietnam War. Having refused to train US Special Forces medics on the grounds that they would use that medical knowledge in the prosecution of the “hearts and minds” element of the war effort (and that this would be in violation of the Geneva Conventions), Levy was sentenced to three years in military prison. (Strassfeld 1994).

The treatment of members of one’s own military force can sometimes also cause ethical conflict for a clinician. In some instances, this can be no more challenging than the situation faced by a civilian occupational physician who both works for a company and provides healthcare to its employees; the problems of “dual loyalty” (alternatively termed “mixed agency”) listed in Table 2, such as limits on confidentiality, and incorporating undesired occupational consequences into a patient’s management plan, are little different. However, armed conflict accentuates the magnitude of these problems. Should a doctor certify a reluctant soldier “fit to fight” in the knowledge that this will expose them to a risk of death? Countless doctors during the World Wars thought the answer was “yes”, although they reported moral conflict in doing so (Jones 2008) as have military doctors (reportedly against their own medical judgement) in contemporary conflict. (Rochon 2015) Should a doctor prescribe medications with the intention of enhancing the combat ability of the fighting force, even in the knowledge that such medications (such as stimulants, (Eliyahu et al. 2007), anabolic steroids, unproven vaccines [e.g. anthrax during the first Gulf War], and experimental nerve protectant agents [e.g. pyridostigmine during the first Gulf War] (Fulco et al. 2000; Sidel and Levy 2003) might have adverse effects on individuals? Even if the effectiveness and adverse effect profile of vaccinations are known to be safe, is it acceptable for physicians to be part of a system that compels individuals to be immunised? These historical examples suggest the answer has often been “yes”, although whether these would be repeated today is questionable. In retrospect, the Anthrax vaccination program was found by the US Government to have been “well intentioned but overwrought … As a healthcare effort, the program (compromised) the practice of medicine to achieve military objectives”. (Sidel and Levy 2003).

Scarcity of medical resources in wartime has also compromised medical ethics. US authors point to the example of allocation of inadequate quantities of penicillin in North Africa during the Second World War. Rather than treat seriously wounded soldiers with little prospect of returning to battle, but a greater clinical need, US military physicians chose instead to treat less unwell patients including those with venereal disease. (Sidel and Levy 2003) In perhaps the most extreme violation of conventional medical ethics due to perceived medical necessity, medical officers have facilitated battlefield euthanasia, as documented in conflicts from biblical times to modern Iraq and Afghanistan. (Neuhaus 2011).

Ethical problems for military clinicians are not confined to wartime. Medical knowledge has potential value to those who develop weapons, as well as scientists charged with reducing the harmful effects of weapons on human targets. Neither situation is ethically straightforward. It is appealing to argue that weapons development is contrary the fundamental principles of a medical practitioner, who must “first of all do no harm”. While clearly true if attempting to increase the lethality of kinetic munitions, should it be ethically acceptable for a physician to participate in research into non-lethal alternatives, designed to reduce rather than increase casualties? This is no longer a theoretical question, with reports of research programs investigating use of inhaled medications that might be used, for example, to incapacitate terrorists safely during sieges. Such research is arguably banned by the 1993 Chemical Weapons Convention, (OPCW 1993) to which 193 nations are committed. Medical organisations such as the British Medical Association have explicitly stated that the use of medications as a method of warfare or law enforcement, and research related to this topic, is contrary to good medical practice. (Kmietowicz 2007) Other, more sophisticated non-lethal biological and physical weaponry is in advanced development (for example, a directed-energy weapon that heats the skin through clothing, producing at most blistering, with the intent of dispersing crowds (Gross 2010)), with similar international opinions that doctors should not be involved. (Gross 2010) Can the same be said for medical involvement in developing defences against weaponry and other health threats? On the one hand, this is an extension of a clinician’s role in reducing suffering, applied at a population level (akin to public health) rather than to an individual patient. Conversely, such work potentially augments combat power – contravening a clinician’s role as a non-combatant. This question receives less attention in the published literature, but given the size of many nations’ investments in military health research (e.g. the US Military Health System invested US$2.3 billion in Research, Development, Testing, and Evaluation in 2020 (‘FY2021 Budget Request for the Military Health System’ 2020)), in practice the answer is that this work appears unequivocally supported.

## Four benefits of a non-combatant medical system over one committed only to the support of its own military force (Table 3)

**Table 3:** Four benefits of preserving non-combatant status for clinicians

Benefit
Facilitation of optimal international relationships during the conflict and in the post-conflict state
Freedom of action in accessing a host nation population when military force is unsuitable or unsuccessful – as long as medical care is not made contingent upon host nation support
Justification for prohibition of targeting medical facilities, even if they are of benefit to a military force
Adherence to the same professional standards as civilian clinicians, allowing military clinicians to remain part of this group

Not all historical examples are of conflicts between military and medical ethics. History also provides examples of the benefits that accrue to a nation when humanitarian principles are respected. During the Falklands War, Surgeon-Commander Rick Jolly commanded the Role 2 hospital established at Ajax Bay in support of 3 Commando Brigade Royal Marines. In accordance with Article 10 of Additional Protocol I and Article 7 of Additional Protocol II of the Geneva Conventions, he and the hospital treated both Argentinian and British casualties. (Jolly 1984) Following the war, the Argentinian Government appointed him an Officer in the *Orden de Mayo* in recognition of this service, accompanying his appointment as an Officer of the Order of the British Empire – the only officer to be decorated by both sides of the war. He later spoke of this experience: “Our attitude was simple: to treat the injured Argentinians in a way we would like to be treated. Before the battle of Trafalgar, Nelson wrote a prayer in his cabin, saying: ‘May humanity after victory be the predominant feature in the British Fleet’. As a naval officer those words meant a lot to me, so looking after the enemy’s wounded as though they were your own was instinctive. People assume you’ve got to hate your enemy but that couldn’t be further from the truth. The only people who know what you’re going through are the people on the other side. Over the years I’ve been asked what I’d do if I had to choose who to treat first, an Argentinian or a Brit. My answer was always whoever needed attention more urgently. As far as I am concerned you have to be able to look into your soul and like what you find there”. (Payne and Dagnell 2012).

The Falklands example is one of many that illustrate that while armed conflict typically lasts only a few months or years, nations must find ways to work with one another in the decades that follow. The basis upon which these future relationships is built is often disproportionately influenced by the anecdotal impression of the enemy’s character, conveyed in stories from the time of the conflict. German military surgery was recognised as highly advanced during the First World War, and German military surgeons were noted as having treated British and French prisoners of war without distinction. (Zischek, Grunwald, and Engelhardt 2018) In her diary “Last night I dreamt of peace”, (Tram 2008) North Vietnamese doctor Dang Thuy Tram recounts her experience as a newly-graduated surgeon working on the Ho Chi Minh trail during the Vietnamese-American war in an idealistic manner that elicits the sympathy of her reader, in so doing humanising the enemy for whom she fought. Whether or not these stories were representative, they served the purpose of reconciling former combatants to working together in the necessary post-conflict reconstruction phase.

The Additional Protocol I of the Geneva Conventions defines combatants as “members of the armed forces of a Party to a conflict (who) have the right to participate directly in hostilities”. (ICRC 1977a) Non-combatants must not participate directly in hostile acts, but they are not prohibited from acting in other ways to the benefit of their own side. As long as healthcare is freely given and not contingent upon support, it is a perfectly acceptable role for military clinicians. In many historical examples, provision of healthcare has enabled access to a host nation population and achievement of military objectives when military force alone had not succeeded. A recent example – albeit one that has not outlived events of August 2021 – is the work of the Provincial Reconstruction Teams (PRTs) in Afghanistan, which were military – led interagency organisations that aimed to enhance the legitimacy of the Government of the newly established Republic of Afghanistan. (Brown 2007) While not exclusively based around healthcare interventions, PRTs often developed health infrastructure and facilitated public health interventions. The emphasis of the PRT was to build host nation capacity and to strengthen local systems of governance, not to directly facilitate military operations by, for example, gathering intelligence or gaining access to the local population. If undertaken well, embedding non-combatants in such organisations could have been entirely consistent with the principles underlying the Geneva Conventions. It is important to note that the use of clinicians in such operations is sometimes erroneously termed “humanitarian”. The four “humanitarian principles” are humanity, neutrality, impartiality and independence, and while such assistance can certainly be given impartially and for human benefit, it is given to meet the aims of the donor as well as the recipient and is clearly associated with national interest.

The third benefit of a non-combatant medical system is that this provides clear justification for prohibiting any form of targeting by armed forces. Whereas a partisan medical system is demonstrably aiding the war effort and therefore targetable, one that is treating all patients according to clinical need alone must surely be respected. The extremely negative public reaction to (claimed) accidental and deliberate attacks on hospitals in Afghanistan, Pakistan, and elsewhere (Benton and Atshan 2016) suggests that armed forces should do everything practical to avoid attacks on what the Western public (at least) expects will be safe spaces.

The fourth benefit of maintaining a non-combatant clinical service is that this allows military clinicians to adhere fully to the same professional ethics as their civilian counterparts. In the Australian context, the Australian public rightly expects the same ethical (and other professional) standards of its military doctors as it does of all Australian doctors and other clinicians. If military clinicians were to be compelled by their role to treat certain patients differently, or to use their position and skill for their own advantage or that of their employer, they would rightly be excluded from their professional clinical communities. One could even then dispute their claim to the title “doctor”, “nurse”, etc. The benefit to the Australian military of avoiding such a situation should be obvious.

## Alternatives to maintaining non-combatants within the military?

One approach to the problems outlined here might be to remove medical and related staff entirely from a military organisation – as has been seriously advocated by some. (Sidel and Levy 2003) All uniformed military personnel would then be combatants with an unambiguous purpose. In its most extreme form, this might involve a return to reliance on volunteer organisations such as existed before and during the First World War, presumably in the form of modern non-government organisations. However, even non-government organisations must receive funding from somewhere – in 2020, 94.1% of the CHF1.87 billion (A$2.8 billion) funding for the International Committee of the Red Cross came from national governments, (ICRC 2020) suggesting that such an arrangement would not free clinicians from partisan allegiance. Furthermore, without military operational control over such organisations during conflict, the likelihood of safe and efficient service delivery would substantially diminish.

It therefore seems likely that modern military forces will retain direct control over the integrated deployable healthcare elements that provide them support. The question becomes how to navigate the issues identified in this paper most effectively.

## Maintaining the balance between medical and military ethics

It is fairly straightforward (for most, if not all (Sidel and Levy 2003)) to emerge from classroom discussions with a clear idea of how a military clinician should act when confronted with various ethical dilemmas, applying the over-riding principles of non-combatant status, understanding the requirements of International Humanitarian Law, and imagining that there should be no difficulties when these are translated to real-life situations. However, as Messelken recognised, “The blurring of military and medical roles is particularly problematic when it is ultimately the responsibility of the individual military doctor to weigh up the roles against each other – if need be, even on a situational basis. Discussions indicate that military doctors with little experience, or ones who are stationed in combat situations, in some cases suppress their medical ethical and legal obligations and perceive themselves (primarily or exclusively) as soldiers. Group dynamics in small units can amplify this tendency”. (Messelken 2015) What can an institution do to prevent situations such as that which occurred with Dr Keilloh in Iraq in 2003?

The most important step towards this goal is the realisation that neither the military nor medical ethic has the claim to moral superiority. As Rascona points out, “Society labels the deaths of soldiers in the endeavor of war as the supreme manifestation of duty, honor, and sacrifice”, and further, “The notion that medical ethics may be somehow superior to (all) others, including just war doctrine, would seem to be at the heart of the problem”. (Sidel and Levy 2003) The “problem” alluded to is, in essence, that most texts discussing this subject are written from the perspective of a doctor wrestling with the challenges of military obligations, making the implicit assumption that medical ethics are those that should be accorded primacy. Few combatant officers write from the contrary perspective, yet recognising that their military ethical viewpoint is no less valid is the first step in reaching a common understanding.

Based upon this thought, several practical suggestions can be made. First, the non-combatant role and obligations of military clinicians must be understood throughout the organisation, and reinforced by every level of operational command. Operational planners must take steps to ensure military clinicians are not left professionally isolated, and senior medical officers must have sufficient expertise, influence and visibility of subordinates to provide effective support to those clinicians most likely to be engaged in ethically challenging situations. Ideally, specific written national policy should codify international agreements, so that these can be most effectively taught in military schools and implemented in national law. Mechanisms to identify deviations from acceptable practice must be implemented, ideally in the context of a global clinical continuous quality improvement framework that prioritises improvements rather than apportioning blame.

## Conclusion

The title of this paper asks “whose side are you on?”. To a combatant officer, a clinician who treats both friend and enemy alike, and who cannot participate in hostile actions against the enemy, might indeed appear not to be on the same side. However, not being a combatant party to a conflict does not imply that the non-combatant clinician cannot act in the national interest. The effects of these actions might be less immediately apparent, but they are no less real, and history shows they can be remembered long after the details of the conflict are forgotten. Non-combatant clinicians in our armed forces are unequivocally on our side, as long as our side stands for the good of humanity.
